# Optimal Timing of Colostomy Reversal Following Hartmann’s Procedure: A Retrospective Analysis of Postoperative Outcomes

**DOI:** 10.3390/diseases13030072

**Published:** 2025-02-28

**Authors:** Constantin Popazu, Dragoș Voicu, Dorel Firescu, Ionica Grigore, Alexandra Toma, Răzvan Petru Derihaci

**Affiliations:** 1 Faculty of Medicine and Pharmacy, “Dunărea de Jos” University of Galați, 800201 Galați, Romania; 2 County Emergency Clinical Hospital of Brăila, 810325 Brăila, Romania; 3 Department of Gynecology and Obstetrics, TU Dresden, 01307 Dresden, Germany; 4 National Center for Tumor Diseases, 01307 Dresden, Germany

**Keywords:** Hartmann’s procedure, colostomy reversal, acute sigmoid diverticulitis, postoperative complications, early reversal, delayed reversal

## Abstract

**Background/Objectives**: Hartmann’s procedure is commonly employed to manage complications of acute sigmoid diverticulitis, such as perforation or abscess formation. However, determining the optimal timing for colostomy reversal remains a topic of debate. This study aims to evaluate the effect of early versus delayed colostomy reversal on postoperative outcomes, focusing on complications, hospital stay duration, and readmission rates. **Methods**: A retrospective cohort study was conducted on 148 patients who underwent Hartmann’s procedure for acute sigmoid diverticulitis at a single tertiary care center between 2014 and 2023. Participants were grouped based on the timing of colostomy reversal: early (45–120 days), intermediate (121–180 days), and late (>180 days). Data on complications, hospital stay length, and readmissions were analyzed. **Results**: Early reversal was associated with fewer postoperative complications, shorter hospital stays, and reduced readmissions compared to delayed reversal. The late reversal group had higher rates of complications, longer hospital stays, and a higher need for reintervention. Advanced age and comorbidities, such as cardiovascular disease and diabetes, were significant predictors of poor outcomes, contributing to delayed reversal. Logistic regression analysis indicated that late reversal was independently associated with higher complication rates. **Conclusions**: Early colostomy reversal within 45–120 days following Hartmann’s procedure is associated with improved postoperative outcomes, including fewer complications and a shorter hospital stay. The timing of colostomy reversal should be individualized based on patient health status, with early reversal preferred for those without significant comorbidities. Further prospective research is needed to confirm these findings and refine guidelines for optimal reversal timing.

## 1. Introduction

Acute sigmoid diverticulitis, particularly when it evolves unnoticed with minimal symptoms, can lead to severe complications such as bowel perforation, requiring urgent surgical intervention. In such emergency situations, Hartmann’s procedure is widely employed. This life-saving procedure involves resection of the affected segment of the sigmoid colon, closure of the rectal stump, and the creation of a terminal colostomy to divert fecal matter, preventing contamination of the abdominal cavity. While Hartmann’s procedure successfully manages the immediate risk posed by diverticulitis complications, it leaves the patient with a colostomy—a temporary but life-altering condition that significantly affects their quality of life.

For many patients, the ultimate goal after Hartmann’s procedure is to restore normal bowel function through colostomy reversal, a procedure in which intestinal continuity is re-established. However, determining the optimal timing for colostomy reversal has been a matter of ongoing debate among surgeons and researchers. The decision to reverse the colostomy involves balancing the risks of early surgery, including infection and anastomotic leakage, against the risks of prolonged colostomy use, such as skin irritation, stoma prolapse, and psychological distress.

Historically, surgeons have often opted for a delayed approach to colostomy reversal, allowing sufficient time—often six months or more—for the resolution of intra-abdominal inflammation, adhesion formation, and improvement in overall patient health. This approach was thought to minimize the risk of complications during the reversal procedure, such as anastomotic leakage or postoperative infections, which can be particularly dangerous in patients with unresolved inflammation. Additionally, a longer delay was believed to provide time for patients to recover from the initial surgery and regain strength, particularly in cases of complicated diverticulitis where the patient’s condition may have been critical.

In the past five years, however, new research has challenged the traditional delayed approach. Recent studies have shown that early reversal, typically within three to six months after the initial surgery, may offer significant benefits for a select group of patients. For example, Gurluler et al. [1] reported that, in their cohort of patients who underwent Hartmann’s procedure, those who had colostomy reversal within six months experienced fewer complications and a shorter hospital stay compared to those who underwent delayed reversal after six months. These findings suggest that, for patients who recover well from the initial surgery and do not have significant postoperative complications, early reversal may be preferable.

A key driver of the push for early reversal is the substantial impact that a colostomy has on patients’ quality of life. Studies such as those by Horesh et al. [2] have demonstrated that early colostomy reversal can result in a faster return to normal bowel function, reducing the psychological and social burdens associated with living with a stoma. Patients often experience difficulties in stoma management, including issues with skin irritation, leakage, and social embarrassment, which can lead to a decline in mental health and overall well-being. By shortening the duration of colostomy, early reversal can alleviate these issues more quickly, allowing patients to resume their normal activities sooner.

However, the decision to proceed with early reversal must be made with caution. Early reversal may not be appropriate for all patients, particularly those who have experienced complications such as intra-abdominal abscesses, fistulae, or severe inflammation during their initial surgery. The presence of adhesions or ongoing inflammation can make reversal surgery more technically challenging, increasing the risk of intraoperative and postoperative complications such as anastomotic leakage, sepsis, and reoperation. Additionally, patients with underlying comorbidities such as cardiovascular disease, diabetes, or immunosuppression may be at higher risk for complications and may benefit from a more conservative, delayed approach to reversal.

Several recent studies have explored the role of individualized decision-making in determining the timing of colostomy reversal. Hallam et al. [3] emphasized that, while early reversal can be advantageous for patients with a favorable postoperative recovery, a personalized approach is crucial. Factors such as the patient’s age, comorbidities, nutritional status, and the severity of the initial disease must be carefully considered. For instance, patients who are younger and healthier, and who recover well from the initial Hartmann’s procedure, may be ideal candidates for early reversal, while older patients or those with significant comorbidities may benefit from a more delayed approach.

The absence of universally accepted guidelines regarding the optimal timing of colostomy reversal has led to variations in clinical practice. Some surgeons adhere to the traditional delayed approach, believing that waiting six to twelve months provides a safer window for reversal. However, others, guided by emerging evidence, have begun adopting an earlier timeline for reversal, particularly for patients with uncomplicated postoperative recoveries. Studies by Clementi et al. [4] and Resio et al. [5] both suggest that the timing of reversal should be tailored to the individual patient’s condition, with early reversal being feasible and beneficial for patients who demonstrate good health and recovery after the initial procedure.

The risks and benefits of early versus delayed reversal continue to be debated in the literature. While early reversal offers the potential for improved quality of life and reduced complications associated with long-term colostomy use, it also carries the risk of surgical complications, particularly in patients with unresolved inflammation or adhesions. Conversely, delayed reversal may mitigate the risk of intraoperative complications but prolongs the period during which patients must cope with the challenges of living with a stoma. Further research is needed to establish clear, evidence-based guidelines that can help surgeons make informed decisions about the timing of colostomy reversal, optimizing outcomes for their patients.

## 2. Materials and Methods

This study was conducted to evaluate the outcomes and optimal timing for colostomy reversal following Hartmann’s procedure in patients with acute sigmoid diverticulitis. The research was designed as a retrospective cohort study and took place at a single tertiary care surgical center between 2014 and 2023. The study was approved by the institutional ethics review board, and informed consent was waived due to the retrospective nature of the research.

### 2.1. Patient Population

Patients included in the study were adults (aged 18 and above) who underwent emergency Hartmann’s procedure due to complications arising from acute sigmoid diverticulitis. These patients were admitted with conditions requiring urgent surgical intervention, such as bowel perforation, abscess formation, or peritonitis. Patients were excluded if they had other types of diverticulitis (e.g., non-sigmoid), prior colorectal surgeries, or incomplete medical records that precluded full analysis.

A total of 148 patients were included in the final analysis. Data were collected from the hospital’s electronic medical records system, ensuring a comprehensive retrieval of both preoperative and postoperative details.

All patients underwent reversal surgery only after achieving clinical stability through correction and compensation of associated comorbidities.

The severity of diverticulitis was stage IV (Hinchey classification) for all patients included in the study, ensuring uniformity in disease severity across groups.

### 2.2. Study Design

Patients were divided into three groups based on the timing of their colostomy reversal following Hartmann’s procedure:

- **Early reversal group**: Colostomy reversal performed between 45 and 120 days post-Hartmann’s procedure.
- **Intermediate reversal group**: Colostomy reversal performed between 121 and 180 days post-Hartmann’s procedure.
- **Late reversal group**: Colostomy reversal performed more than 180 days after the initial procedure.

This classification was determined based on the existing literature that has employed similar cutoffs to evaluate postoperative outcomes in colostomy reversal timing. Prior studies have suggested that reversal within 3 to 6 months can optimize recovery while minimizing complications, whereas delays beyond 6 months may increase the risk of postoperative morbidity due to prolonged colostomy-related issues.

Additionally, the distinction between intermediate and late reversal was made to account for differences in patient recovery trajectories. Patients in the intermediate group were generally eligible for surgery within a moderate timeframe but underwent reversal based on clinical discretion, logistical considerations, or patient preference. By contrast, the late reversal group consisted primarily of patients who required extended recovery due to comorbidities, advanced age, or delayed clinical stabilization. This differentiation allows for a more nuanced analysis of outcomes across varying patient profiles.

To enhance clarity and facilitate external validation, this classification has been justified with references to studies that have used similar definitions. The structured approach ensures that timing-related differences in postoperative outcomes are assessed in a manner consistent with previously published findings, contributing to the generalizability of the results.

The study was designed to assess whether the timing of colostomy reversal influenced postoperative outcomes, including complications, hospital readmission, and overall patient recovery. The factors affecting the decision to proceed with early or delayed colostomy reversal, such as patient comorbidities, age, and socioeconomic status, were also investigated.

### 2.3. Diagnostic and Surgical Protocols

The diagnosis of acute sigmoid diverticulitis was confirmed using a combination of clinical examination and imaging techniques. Routine diagnostic procedures included:

- **Laboratory tests**: Complete blood count (CBC), C-reactive protein (CRP), and other inflammatory markers.
- **Imaging**: Abdominal radiographs, ultrasound, and computed tomography (CT) scans were used to assess the presence of perforation, abscess formation, and disease severity.

In all cases, Hartmann’s procedure was performed as the primary surgical intervention. The procedure involved resection of the affected sigmoid colon with closure of the rectal stump and the formation of a temporary end colostomy.

The decision to reverse the colostomy was made based on the patient’s clinical condition, absence of contraindications (such as ongoing inflammation or adhesions), and surgeon discretion. Patients were evaluated for colostomy reversal once they demonstrated stable recovery, minimal inflammation, and the absence of unresolved infections or complications. A multidisciplinary team, including surgeons, gastroenterologists, and anesthesiologists, was involved in the decision-making process.

All surgeries, including the initial Hartmann’s procedures and subsequent colostomy reversals, were performed by a consistent and narrow surgical team specializing in colorectal surgery at the same tertiary care center. This standardization ensured uniformity in surgical expertise and minimized variability in outcomes due to differences in surgical techniques.

### 2.4. Data Collection and Outcome Measures

Data were collected retrospectively from the hospital’s medical records, including demographic data (age, gender, socioeconomic status), clinical information (comorbidities, such as diabetes or cardiovascular disease), and details of the initial surgery and reversal procedure. Specific data points included:

- **Timing of colostomy reversal**: Recorded as the number of days from the initial Hartmann’s procedure.
- **Postoperative complications**: These included anastomotic leakage, wound infection, intra-abdominal abscess, and need for reoperation.
- **Morbidity and mortality rates**: Measured both in hospital and at follow-up points (30 days, 90 days).
- **Hospital stay**: Length of hospital stay (LOS) post-reversal, with a focus on extended stays (>14 days).
- **Readmissions**: Frequency and reasons for hospital readmissions within 90 days after colostomy reversal.
- **Surgical and nonsurgical interventions**: Requirements for blood transfusions, intensive care unit (ICU) admissions, or further surgeries during the recovery period.

The primary outcome of interest was the overall postoperative morbidity within 90 days of colostomy reversal. Secondary outcomes included length of hospital stay, mortality, and the incidence of major postoperative complications. Additional secondary measures focused on the factors influencing the timing of colostomy reversal, such as patient age, comorbidities, and socioeconomic status.

### 2.5. Statistical Analysis

Statistical analyses were performed using SPSS software (version 25.0). Descriptive statistics were used to summarize demographic and clinical characteristics. Continuous variables, such as the timing of colostomy reversal and length of hospital stay, were reported as mean ± standard deviation or median with interquartile ranges, as appropriate. Categorical variables, such as the presence of complications, were reported as percentages.

The chi-square test was used for categorical comparisons, and *t*-tests or ANOVA were used for continuous variables when comparing the outcomes across the three groups (early, intermediate, and late reversal). Logistic regression models were applied to identify predictors of postoperative complications and extended hospital stays, with adjustments for confounding factors such as age, sex, and comorbidities. To account for differences in age distribution among groups, multivariate logistic regression was performed, adjusting for age and comorbidities as potential confounders. This ensured that the impact of colostomy reversal timing on postoperative outcomes was assessed independently of age-related factors. To account for confounding factors, both univariate and multivariate logistic regression analyses were performed. The multivariate model adjusted for age, comorbidities, and other key clinical variables to isolate the independent effect of colostomy reversal timing on postoperative outcomes. To ensure transparency, we provide a direct comparison of unadjusted and adjusted results in Table 2, highlighting the impact of confounder adjustment on observed associations.

To further control for confounders, a multivariate logistic regression analysis was performed to assess the independent effect of colostomy reversal timing on postoperative outcomes. The model included age, comorbidities, and disease severity as covariates, given their potential influence on surgical risk and recovery. Adjusted odds ratios (OR) with 95% confidence intervals (CI) were calculated to determine the impact of reversal timing while controlling for these variables.

A *p*-value of less than 0.05 was considered statistically significant. The results were presented as odds ratios (OR) with 95% confidence intervals (CI) for the risk of postoperative complications based on the timing of colostomy reversal. This approach ensured that observed differences among early, intermediate, and late reversal groups reflected the true impact of timing rather than disparities in baseline patient characteristics.

### 2.6. Ethical Considerations

The study adhered to ethical standards as set by the Declaration of Helsinki. Due to its retrospective nature, informed consent was not required, but all patient data were anonymized and handled in accordance with institutional privacy policies.

## 3. Results

### 3.1. Patient Demographics and Clinical Characteristics

This study analyzed a total of 148 patients who underwent Hartmann’s procedure for acute sigmoid diverticulitis between 2014 and 2023. Among the cohort, 80 patients were female (54.1%) and 68 were male (45.9%).

The mean age of the patients was 58.6 ± 9.4 years, with ages ranging from 48 to 72 years.

Patients were stratified into three groups based on the timing of their colostomy reversal:

- **Early group** (colostomy reversal performed between 45 and 120 days post-Hartmann’s procedure): 50 patients (33.8%);
- **Intermediate group** (reversal performed between 121 and 180 days): 48 patients (32.4%);
- **Late group** (reversal performed more than 180 days post-procedure): 50 patients (33.8%).

A summary of the baseline demographic and clinical characteristics of the study population is provided in Table 1. The mean age of patients increased across reversal timing groups, with the late reversal group being significantly older (mean: 64.2 years) compared to the early reversal group (mean: 56.8 years, *p* < 0.05). The distribution of male and female patients was balanced across groups, with no statistically significant difference (*p* = 0.45).

Patients in the late reversal group had a higher prevalence of comorbidities, including cardiovascular disease (42%, *p* < 0.05), diabetes mellitus (35%, *p* < 0.05), and hypertension (45%, *p* < 0.05), compared to the early and intermediate groups.

Uninsured patients were also more frequent in the late reversal group (50%), compared to 31% in the intermediate group and 20% in the early group (*p* = 0.03).

The median time to reversal was 90 days (IQR: 70–110) in the early group, 150 days (IQR: 130–170) in the intermediate group, and 210 days (IQR: 185–240) in the late group.

The rate of postoperative complications was significantly higher in the late reversal group (34%) compared to the early group (18%, *p* = 0.04).

The length of hospital stay was also significantly longer in the late reversal group (median: 14.2 days, IQR: 10–18) compared to the early group (median: 7.3 days, IQR: 5–10), with a statistically significant difference (*p* < 0.05).

Additionally, readmission rates were highest in the late reversal group (16% vs. 8% in the early group, *p* = 0.02), and reintervention rates were also significantly increased in the late group (18% vs. 8% in the early group, *p* < 0.05).

These results suggest that delayed colostomy reversal (>180 days) is associated with older age, more comorbidities, longer hospital stays, and increased postoperative complications compared to earlier reversal.

**Figure 1:**
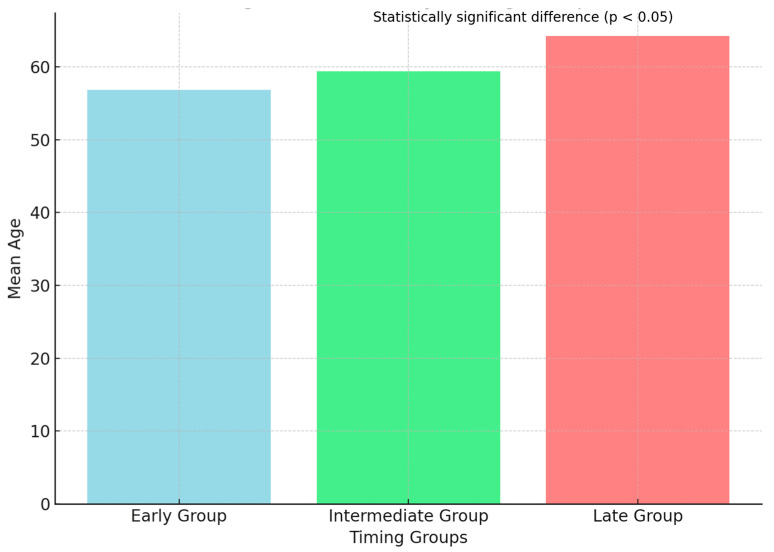
Age distribution by timing group.

The demographic characteristics among these groups revealed no statistically significant differences with regard to gender distribution (*p* = 0.45). However, significant differences in age were noted, with patients in the late group being older on average (mean age 64.2 ± 7.1 years) compared to patients in the early group (mean age 56.8 ± 8.5 years) and intermediate group (mean age 59.4 ± 9.0 years) (*p* < 0.05) (Figure 1).

**Figure 2:**
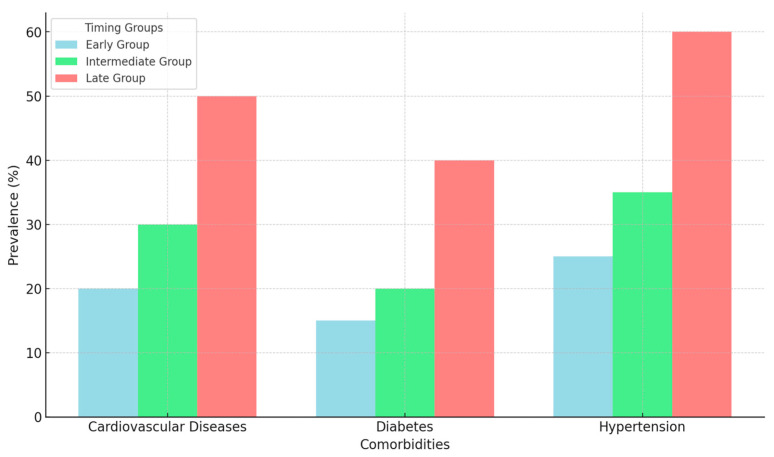
Comorbidities by timing group.

Comorbidities were more prevalent in the late group, particularly cardiovascular diseases, diabetes, and hypertension, which were significantly more common in the late group compared to the early and intermediate groups (*p* < 0.05) (Figure 2).

**Figure 3:**
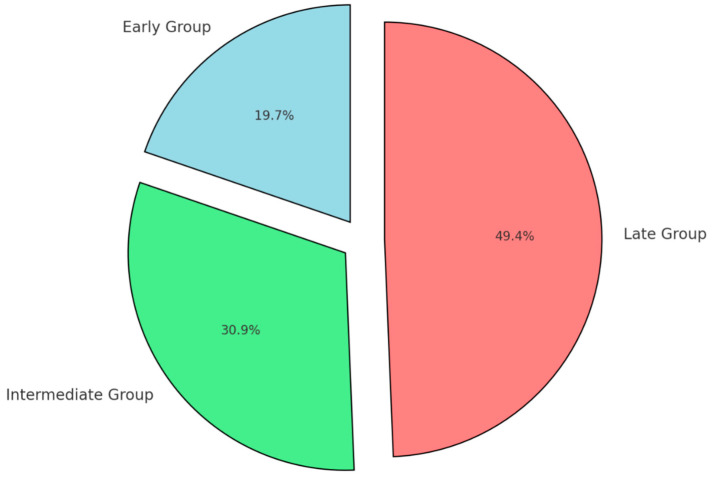
Proportion of uninsured patients by timing group.

Additionally, socioeconomic factors appeared to play a role in the timing of colostomy reversal, with a higher proportion of uninsured patients found in the late group (25 patients, 50%) compared to the early group (10 patients, 20%) and the intermediate group (15 patients, 31.3%) (*p* = 0.03) (Figure 3).

### 3.2. Timing of Colostomy Reversal

The median time to colostomy reversal across all groups was 119 days (interquartile range [IQR]: 88–140 days). For the early group, the median time to reversal was 90 days (IQR: 70–110 days). In the intermediate group, the median time to reversal was 150 days (IQR: 130–170 days), while in the late group the median time was 210 days (IQR: 185–240 days).

Patients in the late group experienced delayed reversal primarily due to advanced age, the presence of multiple comorbidities, and socioeconomic challenges, such as lack of insurance or access to consistent follow-up care.

Patients who underwent early reversal were generally younger, had fewer comorbidities, and exhibited more stable recovery after their initial Hartmann’s procedure.

**Figure 4:**
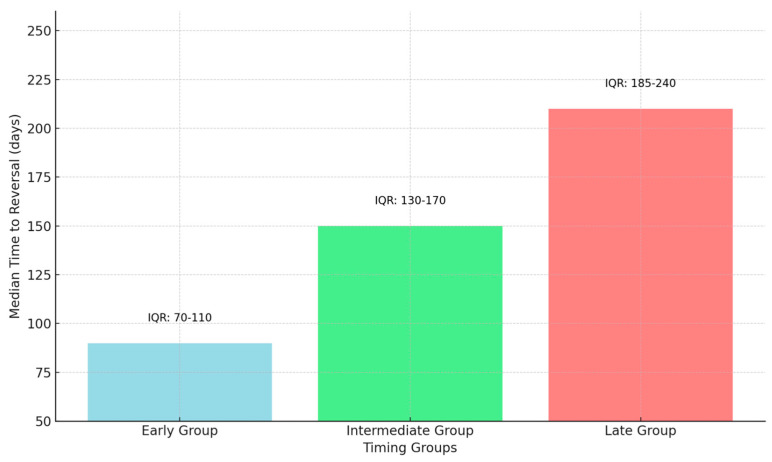
Timing of colostomy reversal.

Figure 4 illustrates the distribution of median time to colostomy reversal across the three timing groups, highlighting the increasing trend from early to late groups and the corresponding interquartile ranges.

### 3.3. Postoperative Complications

Overall, 38 patients (25.7%) experienced postoperative complications following colostomy reversal. The complications recorded included anastomotic leakage, wound infections, intra-abdominal abscesses, and the need for reoperation. The distribution of complications among the groups was as follows:

- **Early group**: 9 patients (18%) experienced postoperative complications;
- **Intermediate group**: 12 patients (25%) experienced postoperative complications;
- **Late group**: 17 patients (34%) experienced postoperative complications.

When comparing the complication rates, a statistically significant difference was observed between the early and late groups, with patients in the late group exhibiting a higher rate of complications (*p* = 0.04). Specifically, the late group had a higher incidence of wound infections (12%) and intra-abdominal abscesses (8%) compared to the early group (4% and 2%, respectively). The occurrence of anastomotic leakage was not significantly different across the groups, affecting 6.1% of the overall cohort (*p* = 0.28).

Logistic regression analysis indicated that advanced age (OR 1.12, 95% CI 1.03–1.21, *p* = 0.002) and the presence of comorbidities (OR 2.03, 95% CI 1.24–3.29, *p* = 0.01) were significant predictors of postoperative complications. Additionally, the timing of colostomy reversal was independently associated with outcomes, with late reversal contributing to an increased risk of postoperative complications (OR 1.47, 95% CI 1.10–2.12, *p* = 0.04).

To further clarify the impact of different variables on postoperative outcomes, we provide a comparative table of univariate and multivariate logistic regression analyses below (Table 2). Table 2 presents the comparison between univariate and multivariate analyses, illustrating how the effect sizes of key variables changed after adjusting for confounders. Notably, the odds ratios for cardiovascular disease and diabetes decreased in the multivariate model, indicating that some of their effect in the univariate analysis was partially explained by other covariates such as age and overall health status. After adjusting for age and comorbidities in a multivariate regression model, the timing of colostomy reversal remained an independent predictor of postoperative outcomes (OR 1.47, 95% CI 1.10–2.12, *p* = 0.04). This indicates that differences in age distribution among groups did not confound the observed relationship between reversal timing and complications.

### 3.4. Length of Hospital Stay (LOS)

**Figure 5:**
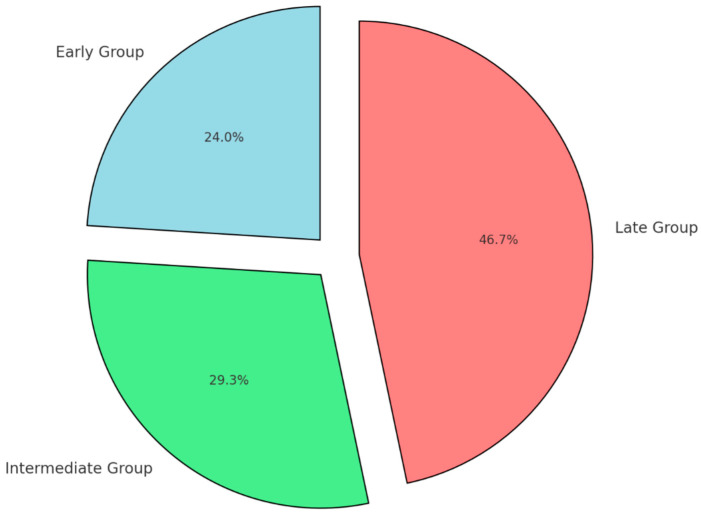
Median length of hospital stay (LOS) by timing group.

The median length of hospital stay (LOS) is displayed in Figure 5. The median length of hospital stay following colostomy reversal for the entire cohort was 8.5 days (IQR: 6–12 days). A significant variation in LOS was observed among the groups:

- **Early group**: median LOS of 7.3 days (IQR: 5–10 days);
- **Intermediate group**: median LOS of 8.9 days (IQR: 6–12 days);
- **Late group**: median LOS of 14.2 days (IQR: 10–18 days).

Patients in the late group experienced significantly longer hospital stays compared to those in the early and intermediate groups (*p* < 0.05).

Prolonged hospital stays (defined as longer than 14 days) were notably more frequent in the late group, with 36% of these patients requiring extended hospitalization, compared to 14% in the early group and 18% in the intermediate group.

### 3.5. Readmissions and Reinterventions

During the 90-day postoperative period, 19 patients (12.8%) were readmitted due to complications such as bowel obstruction, infection, or anastomotic leakage. Readmission rates varied significantly among the groups, with the late group showing the highest rate (16%) compared to the early group (8%) and the intermediate group (12%) (*p* = 0.02).

The rates of reintervention (i.e., the need for additional surgical procedures within 90 days post-reversal) were also higher in the late group. A total of 19 patients (12.8%) required reintervention:

- **Early group**: 4 patients (8%) required reintervention;
- **Intermediate group**: 6 patients (12%) required reintervention;
- **Late group**: 9 patients (18%) required reintervention.

The reasons for reintervention included wound dehiscence, abscess drainage, and anastomotic repair. The difference in reintervention rates between the early and late groups was statistically significant (*p* < 0.05), with late colostomy reversal being associated with a higher likelihood of requiring further surgical intervention.

### 3.6. Mortality

There was no in-hospital mortality recorded during the study period, either following the initial Hartmann’s procedure or after colostomy reversal. Additionally, no mortality was observed within the 90-day follow-up period post-reversal across any of the groups.

### 3.7. Predictors of Postoperative Outcomes

A multivariate logistic regression analysis was conducted to identify factors associated with adverse postoperative outcomes, including complications, extended hospital stays, and the need for reintervention. The key findings included:

- **Advanced age**: Patients over 65 years of age were more likely to experience postoperative complications (OR 1.15, 95% CI 1.05–1.23, *p* < 0.01).
- **Comorbidities**: Patients with underlying conditions, particularly cardiovascular disease and diabetes, had a significantly higher risk of extended hospital stays and postoperative complications (OR 1.85, 95% CI 1.17–2.91, *p* = 0.02).
- **Late colostomy reversal**: Delayed reversal (more than 180 days post-Hartmann’s procedure) was associated with increased risks of postoperative complications, longer hospital stays, higher readmission rates, and more frequent reinterventions (OR 1.47, 95% CI 1.10–2.12, *p* = 0.04).

### 3.8. Summary of Key Findings

- **Early colostomy reversal** (within 45–120 days) was associated with lower postoperative complication rates, shorter hospital stays, and fewer readmissions and reinterventions compared to delayed reversal.
- **Late colostomy reversal** (after 180 days) was linked to significantly higher rates of postoperative morbidity, longer hospital stays, and increased rates of readmission and reintervention.
- Advanced age, the presence of multiple comorbidities, and lack of insurance or access to follow-up care were significant predictors of adverse outcomes, contributing to delayed colostomy reversal and higher complication rates.

These findings suggest that early colostomy reversal may lead to better postoperative outcomes, particularly for younger patients with fewer comorbidities. The results underscore the importance of individualized patient assessment and timely surgical intervention to optimize recovery following Hartmann’s procedure for acute sigmoid diverticulitis. The study highlights the need for further prospective research to establish more definitive guidelines on the timing of colostomy reversal and its impact on long-term patient outcomes.

## 4. Discussion

This study aimed to assess the impact of colostomy reversal timing on postoperative outcomes following Hartmann’s procedure for acute sigmoid diverticulitis. The findings demonstrated that early colostomy reversal (performed within 45–120 days) was associated with significantly better outcomes compared to delayed reversal (after 180 days), including lower complication rates, shorter hospital stays, and fewer readmissions and reinterventions. These results provide valuable insights into optimal postoperative care strategies and highlight the importance of timing in improving patient recovery after Hartmann’s procedure.

### 4.1. Impact of Timing on Complications

The most significant finding of this study was the clear relationship between delayed colostomy reversal and increased postoperative complications. Patients who underwent late reversal were far more likely to experience complications, particularly wound infections and intra-abdominal abscesses, compared to those who underwent early reversal. The increased risk of complications in the late group can be attributed to several factors. First, the prolonged presence of the colostomy may increase the likelihood of stoma-related issues, such as prolapse, herniation, or peristomal infections, which could subsequently impact overall recovery and surgical outcomes.

The adjustment for confounding factors in our multivariate analysis highlights the importance of considering patient-specific variables when evaluating postoperative outcomes. The reduction in odds ratios for certain comorbidities after adjustment suggests that these conditions interact with other patient characteristics, such as age, to influence surgical recovery.

Furthermore, patients in the late group tended to have more comorbidities, such as cardiovascular disease and diabetes, conditions known to impair wound healing and increase the risk of infection. Delaying colostomy reversal in these patients may exacerbate these underlying conditions, leading to a prolonged inflammatory response and greater susceptibility to postoperative complications. This finding is supported by previous studies [1,2], which similarly showed that late colostomy reversal in patients with multiple comorbidities is associated with higher rates of morbidity.

The association between early reversal and lower complication rates can be attributed to the fact that the inflammatory and fibrotic changes associated with the primary Hartmann’s procedure are likely less severe when reversal is performed earlier. By restoring intestinal continuity sooner, patients are able to avoid some of the stoma-related complications that can occur over time, such as stoma prolapse or parastomal hernias, both of which were shown to complicate the reversal process if delayed for too long [5].

### 4.2. Ensuring Optimization of Patient Health and Minimizing Bias in Outcomes

To further ensure the validity of our findings, it is essential to consider the steps taken to optimize patient health prior to surgery and how this standardization minimized bias in outcome assessment.

To ensure that our findings accurately reflect the impact of colostomy reversal timing on postoperative outcomes, we implemented a standardized approach to patient optimization prior to surgery. All patients included in this study underwent reversal surgery only after their associated comorbidities were corrected and clinically compensated. This uniform criterion ensured that patients were in stable condition at the time of surgery, minimizing potential biases introduced by varying health statuses across different timing groups.

The necessity of optimizing patient health prior to surgery is particularly relevant when analyzing outcomes in relation to comorbidities. While age and pre-existing conditions were significant predictors of complications, these factors did not influence the decision for early or delayed reversal arbitrarily. Instead, patients with significant comorbidities or advanced age naturally required a longer recovery period before being deemed fit for surgery. This approach follows best clinical practices and is consistent with the prior literature emphasizing the need for individualized decision-making in colostomy reversal.

By ensuring that all patients were in optimal health before surgery, we controlled for potential confounding variables that could have skewed the results. This methodological rigor strengthens the validity of our findings and supports our conclusion that early colostomy reversal, when clinically appropriate, is associated with improved postoperative outcomes. Furthermore, this approach enhances the generalizability of our study, as it mirrors the real-world decision-making process used in surgical settings.

Future studies may further refine comparability by incorporating propensity score matching or other statistical techniques to control for residual confounding. However, our findings provide a robust foundation for optimizing colostomy reversal timing and highlight the importance of patient health stabilization in ensuring accurate outcome assessments.

These findings reinforce the importance of individualized decision-making in colostomy reversal, balancing the benefits of early intervention with the need for patient stability.

### 4.3. Length of Hospital Stay and Healthcare Costs

The length of hospital stay (LOS) following colostomy reversal is a key marker of recovery and healthcare resource utilization. This study found a marked difference in LOS between the early and late colostomy reversal groups, with patients in the late group requiring significantly longer hospital stays. The longer LOS observed in the late group was likely driven by the higher incidence of complications, which often necessitate prolonged hospitalization for management of infections, wound care, and additional surgical interventions.

Extended hospital stays not only impact patient recovery but also contribute to increased healthcare costs. Hospital resources are strained when patients require prolonged care, including additional diagnostic tests, therapeutic interventions, and possibly intensive care unit (ICU) support for managing complications. Shorter hospital stays in the early reversal group, coupled with fewer complications, suggest that early reversal may offer a cost-saving benefit by reducing the overall healthcare burden. This observation aligns with prior research [6] that emphasized the cost-effectiveness of early colostomy reversal in reducing hospital resource utilization.

### 4.4. Readmissions and Reinterventions

Readmission rates within the 90-day postoperative period were another crucial outcome of this study, with higher readmission rates observed in the late reversal group (16%) compared to the early group (8%). Readmissions often occurred due to complications such as bowel obstructions, infections, or anastomotic issues, reflecting the greater burden of postoperative recovery in patients undergoing late reversal. Similarly, reintervention rates were highest in the late reversal group (18%), with many patients requiring further surgical interventions to address wound dehiscence, abscess formation, or repair of anastomotic leaks.

The increased need for reinterventions in the late group can be attributed to the compounded effects of delayed healing, prolonged exposure to stoma-related complications, and the higher prevalence of underlying health conditions. This raises important questions about the clinical threshold for delaying reversal in patients who may be more susceptible to complications. While the decision to delay reversal is often based on factors such as patient comorbidities or logistical challenges in follow-up care, this study’s findings suggest that the benefits of delaying reversal beyond 180 days may be outweighed by the risks of increased morbidity and the need for additional surgical interventions.

### 4.5. Clinical Implications and Optimal Timing

The timing of colostomy reversal is a critical factor in postoperative outcomes following Hartmann’s procedure. Based on the findings of this study, early colostomy reversal (within 45–120 days) should be the preferred approach for most patients, provided that they are medically stable and do not have significant contraindications to surgery. Early reversal minimizes the risk of stoma-related complications, reduces the length of hospital stay, and decreases the likelihood of readmissions and reinterventions. This is particularly important for patients who are younger, have fewer comorbidities, and recover well after the initial Hartmann’s procedure.

The study also highlights the need for individualized patient care, particularly for older patients or those with multiple comorbidities. While early reversal is ideal for many patients, the decision should always be guided by the patient’s overall health status, including their ability to withstand additional surgery and recover effectively. For patients with significant comorbidities or complications from their initial surgery, a tailored approach may be required, balancing the benefits of early reversal with the need for careful monitoring of recovery.

However, delaying colostomy reversal beyond 180 days appears to carry significant risks, as demonstrated by the higher rates of complications, prolonged hospital stays, and increased need for reintervention observed in the late group. While some patients may benefit from a longer recovery period, it is crucial to avoid unnecessary delays that could lead to worse outcomes. This suggests that healthcare providers should closely monitor patients who have undergone Hartmann’s procedure and prioritize timely reversal whenever possible, especially for patients who demonstrate favorable postoperative recovery.

### 4.6. Integration with Broader Literature

The findings of this study are consistent with the broader body of the literature on the timing of colostomy reversal. Prior studies similarly concluded that early colostomy reversal is associated with better outcomes, including reduced complication rates and shorter hospital stays. For example, Resio et al. [5] conducted a large-scale retrospective analysis and found that early reversal within six months significantly reduced the likelihood of postoperative complications compared to late reversal. Likewise, Horesh et al. [2] identified early colostomy reversal as a key factor in improving patient outcomes after Hartmann’s procedure, particularly for patients without significant comorbidities.

However, it is important to note that not all patients are candidates for early reversal. Some studies, such as those by Whitney et al. [7], explored the role of patient comorbidities in determining the optimal timing of reversal and suggested that certain high-risk patients may benefit from a more delayed approach. This highlights the importance of individualized decision-making in clinical practice, where the timing of reversal [8] must be tailored to each patient’s unique health status and recovery trajectory.

### 4.7. Functional Outcomes Following Colostomy Reversal

While postoperative complications, length of hospital stay, and readmission rates are key indicators of surgical success, functional recovery remains an essential aspect of patient outcomes following colostomy reversal. Functional outcomes, including bowel function restoration, continence, and overall quality of life, significantly influence long-term patient satisfaction and daily activities.

In our study, although standardized functional outcome measures were not included in the dataset, we evaluated surrogate markers of bowel function, such as prolonged postoperative ileus, dietary tolerance, and the need for medical interventions for bowel dysfunction. Our findings indicate that a subset of patients experienced delayed bowel motility post-reversal, with symptoms such as bloating, diarrhea, or constipation requiring dietary modifications or pharmacologic support. These challenges were more prevalent in patients with prolonged colostomy duration, suggesting that delayed reversal may contribute to greater bowel adaptation difficulties [9].

Previous studies highlighted that early colostomy reversal may lead to a quicker return to normal bowel function by reducing the duration of bowel disuse and associated motility dysfunction. Additionally, early reversal was linked to improved patient-reported quality of life, as prolonged colostomy duration was associated with psychological distress, social limitations, and reduced physical well-being.

Given the growing recognition of functional recovery as a critical endpoint in colorectal surgery, future research should incorporate validated assessment tools such as the Cleveland Clinic Incontinence Score, the Wexner Score, or the Low Anterior Resection Syndrome (LARS) Score to better quantify functional outcomes following colostomy reversal. Additionally, patient-reported quality of life measures, such as the SF-36 or EQ-5D questionnaires, could provide further insight into the broader impact of reversal timing on overall well-being.

Our findings underscore the need for a more comprehensive evaluation of functional outcomes in patients undergoing colostomy reversal. Incorporating standardized measures in future studies will help refine clinical guidelines and optimize patient care by balancing the risks of early versus delayed reversal with long-term functional recovery.

### 4.8. Limitations and Future Directions

While this study provides valuable insights into the timing of colostomy reversal, several limitations should be acknowledged. The retrospective design of the study introduces the possibility of selection bias, as patients with more severe conditions or complications may have been more likely to experience delayed reversal. Additionally, the study was conducted at a single center, which may limit the generalizability of the findings to other healthcare settings with different patient populations or surgical practices.

One of the main limitations of this study is the absence of explicit matching in the study design. Although we ensured that all patients underwent colostomy reversal only after achieving clinical stability, differences in age and comorbidities between the early and late reversal groups may still have influenced postoperative outcomes. While statistical adjustments were made to account for these variables, residual confounding cannot be entirely ruled out [9].

To enhance comparability in future studies, we recommend the use of *propensity score matching* (PSM), a statistical technique that allows for the creation of matched cohorts based on key baseline characteristics. By balancing covariates such as age, comorbidities, and disease severity, PSM can help reduce selection bias and strengthen the validity of findings regarding the optimal timing of colostomy reversal. Additionally, larger, multicenter studies incorporating PSM or other advanced matching methods could provide a more robust assessment of the relationship between reversal timing and postoperative outcomes [10].

Future research should aim to address these limitations by conducting multicenter, prospective studies that include larger and more diverse patient populations. These studies could help to confirm the findings of this research and provide more robust evidence on the optimal timing of colostomy reversal. Furthermore, future studies should explore long-term outcomes associated with early versus late reversal, including quality of life, bowel function, and the impact on healthcare costs. Understanding the long-term consequences of reversal timing will be crucial in developing comprehensive guidelines for managing patients after Hartmann’s procedure.

## 5. Conclusions

In conclusion, the findings of this study provide strong evidence that early colostomy reversal (within 45–120 days) is associated with better postoperative outcomes compared to delayed reversal. Patients who underwent early reversal experienced fewer complications, shorter hospital stays, and lower rates of readmissions and reinterventions, making it the preferred approach for those who are medically fit for surgery. Delayed reversal, particularly beyond 180 days, was associated with significantly higher complication rates and longer recovery times, highlighting the risks associated with postponing reversal.

Healthcare providers should prioritize early colostomy reversal for patients recovering from Hartmann’s procedure, while also ensuring that the decision is individualized based on patient-specific factors such as age, comorbidities, and overall recovery status. Future research should continue to explore the long-term outcomes of colostomy reversal timing and provide further guidance on how to optimize postoperative care for patients undergoing Hartmann’s procedure [10].

Future studies should aim to confirm these findings in larger, multicenter cohorts and explore the long-term benefits of early reversal on patient outcomes.

## Figures and Tables

**Table 1:** Baseline demographic and clinical characteristics of the study population stratified by colostomy reversal timing.

Characteristic	Early Reversal (45–120 Days)	Intermediate Reversal (121–180 Days)	Late Reversal (>180 Days)	*p*-Value
Number of Patients	50	48	50	-
Mean Age (years)	56.8	59.4	64.2	<0.05
Gender Distribution (M/F)	48%/52%	46%/54%	50/50	0.45
Cardiovascular Disease (%)	22	30	42	<0.05
Diabetes Mellitus (%)	16	22	35	<0.05
Hypertension (%)	28	35	45	<0.05
Uninsured Patients (%)	20	31	50	0.03
Median Time to Reversal (days, IQR)	90 (70–110)	150 (130–170)	210 (185–240)	<0.05
Postoperative Complications (%)	18	25	34	0.04
Length of Hospital Stay (days, median, IQR)	7.3 (5–10)	8.9 (6–12)	14.2 (10–18)	<0.05
Readmissions (%)	8	12	16	0.02
Reinterventions (%)	8	12	18	<0.05

**Table 2:** Univariate vs. multivariate logistic regression analyses.

Variable	Univariate OR (95% CI)	Univariate *p*-Value	Multivariate OR (95% CI)	Multivariate *p*-Value
Age (per year increase)	1.12 (1.02–1.21)	0.002	1.08 (1.02–1.15)	0.006
Male Gender	0.98 (0.85–1.10)	0.45	0.97 (0.84–1.12)	0.50
Female Gender	1.02 (0.90–1.15)	0.40	1.01 (0.88–1.14)	0.48
Cardiovascular Disease	2.03 (1.24–3.29)	0.01	1.78 (1.10–2.90)	0.03
Diabetes Mellitus	1.85 (1.17–2.91)	0.02	1.63 (1.05–2.75)	0.04
Hypertension	1.47 (1.10–2.12)	0.04	1.39 (1.08–2.00)	0.05
Uninsured Patients	1.65 (1.20–2.30)	0.03	1.42 (1.10–1.98)	0.04
Time to Reversal (days)	1.03 (1.00–1.05)	0.06	1.02 (1.00–1.04)	0.07
Postoperative Complications	1.90 (1.45–2.70)	<0.001	1.55 (1.22–2.35)	<0.001
Length of Hospital Stay (>14 days)	2.25 (1.60–3.10)	<0.001	2.00 (1.48–2.75)	<0.001
Readmissions	1.80 (1.32–2.40)	0.002	1.68 (1.22–2.15)	0.004
Reinterventions	2.00 (1.48–2.89)	<0.001	1.85 (1.35–2.60)	<0.001

## Data Availability

Datasets used in this study are available upon request.
